# Supplementary material for: Construction of the core competencies training system for thoracic surgery specialist nurses: A mixed-methods study
Source: PLoS One. 2026 Feb 2;21(2):e0339777. doi: 10.1371/journal.pone.0339777 (PMC12863540; doi:10.1371/journal.pone.0339777)
Supplement: S1 File — (ZIP) [file pone.0339777.s001.zip › S1 File. Literature search strategy.docx]

**Literature search strategy**

**Search time:** Up to December 10, 2024

| **Databases** | **Search Expressions** | **Number of Retrieved Articles** |
| --- | --- | --- |
| PubMed | (((((((((Thoracic Surgery[MeSH Terms]) OR (Thoracic Surgery[Title/Abstract])) OR (Surgery, Thoracic[Title/Abstract])) OR (Cardiothoracic Surgery[Title/Abstract])) OR (General Thoracic Surgery[Title/Abstract])) OR (General Thoracic[Title/Abstract])) OR (Pulmonary Surgery[Title/Abstract])) OR (Esophageal Surgery[Title/Abstract])) AND (((((((((Nurse Practitioners[MeSH Terms]) OR (Specialties, Nursing[MeSH Terms])) OR (Nurse Practitioner*[Title/Abstract])) OR (Special*, Nurs*[Title/Abstract])) OR (Nurs* Special*[Title/Abstract])) OR (Nurs* Personnel*[Title/Abstract])) OR (Professional Nurse[Title/Abstract])) OR (Advanced Practice Nurse[Title/Abstract])) OR (Nurs*[Title/Abstract]))) AND ((((((((((Curriculum[MeSH Terms]) OR (education[MeSH Terms])) OR (Curriculum[Title/Abstract])) OR (education[Title/Abstract])) OR (Curricula[Title/Abstract])) OR (Short Term Course*[Title/Abstract])) OR (Course*, Short-Term[Title/Abstract])) OR (training[Title/Abstract])) OR (cultivate[Title/Abstract])) OR (teaching[Title/Abstract])) | 990 |
| Embase | #1 'thorax surgery'/exp OR 'thoracic surgery':ab,ti OR 'surgery, thoracic':ab,ti OR 'cardiothoracic surgery':ab,ti OR 'general thoracic surgery':ab,ti OR 'general thoracic':ab,ti OR 'pulmonary surgery':ab,ti OR 'esophageal surgery':ab,ti  #2 'nurse practitioner'/exp OR 'nursing discipline'/exp OR 'surgery, thoracic':ab,ti OR 'nurse practitioner*':ab,ti OR 'special*, nurs*':ab,ti OR 'nurs* special*':ab,ti OR 'nurs* personnel*':ab,ti OR 'professional nurse':ab,ti OR 'advanced practice nurse':ab,ti OR nurs*:ab,ti  #3 'curriculum'/exp OR 'education program'/exp OR 'education program':ab,ti OR 'training'/exp OR curriculum:ab,ti OR training:ab,ti OR 'education':ab,ti OR curricula:ab,ti OR 'short term course*':ab,ti OR 'course*, short-term':ab,ti OR cultivate:ab,ti OR teaching:ab,ti  #4=#1 AND #2 AND #3 | 1767 |
| Cochrane | #1 MeSH descriptor: [Thoracic Surgery] explode all trees  #2 (Thoracic Surgery):ti,ab,kw  #3 (Cardiothoracic Surgery):ti,ab,kw  #4 (General Thoracic Surgery):ti,ab,kw  #5 (General Thoracic):ti,ab,kw  #6 (Pulmonary Surgery):ti,ab,kw  #7 (Esophageal Surgery):ti,ab,kw  #8 #1 OR #2 OR #3 OR #4 OR #5 OR #6 OR #7  #9 MeSH descriptor: [Nurse Practitioners] explode all trees  #10 MeSH descriptor: [Specialties, Nursing] explode all trees  #11 (Nurse Practitioner*):ti,ab,kw  #12 (Nurs* Special*):ti,ab,kw  #13 (Nurs* Personnel*):ti,ab,kw  #14 (Professional Nurse):ti,ab,kw  #15 (Advanced Practice Nurse):ti,ab,kw  #16 (Nurs*):ti,ab,kw  #17 #9 OR #10 OR #11 OR #12 OR #13 OR #14 OR #15 OR #16  #18 MeSH descriptor: [Curriculum] explode all trees  #19 MeSH descriptor: [Education] explode all trees  #20 (Curriculum):ti,ab,kw  #21 (education):ti,ab,kw  #22 (Curricula):ti,ab,kw  #23 MeSH descriptor: [Education] explode all trees  #24 (Short Term Course*):ti,ab,kw  #25 (training):ti,ab,kw  #26 (cultivate):ti,ab,kw  #27 (teaching):ti,ab,kw  #28 #18 OR #19 OR #20 OR #21 OR #22 OR #23 OR #24 OR #25 OR #26 OR #27  #29 #8 AND #17 AND #28 | 181 |
| CINAHL | S1: MH Thoracic Surgery OR SU Thoracic Surgery OR SU Surgery, Thoracic OR SU Cardiothoracic Surgery OR SU General Thoracic Surgery OR SU General Thoracic OR SU Pulmonary Surgery OR SU Esophageal Surgery  S2: MH Nurse Practitioners OR SU Nurse Practitioner* OR SU Special*, Nurs* OR SU Nurs* Special* OR SU Nurs* Personnel* OR SU Professional Nurse OR SU Advanced Practice Nurse OR SU Nurs*  S3: MH Curriculum OR MH education OR SU Curriculum OR SU education OR SU Curricula OR SU Short Term Course* OR SU Course*, Short-Term OR SU training OR SU cultivate OR SU teaching  S4=S1 AND S2 AND S3 | 42 |
| Web of Science | 1: Thoracic Surgery/Surgery, Thoracic/Cardiothoracic Surgery/General Thoracic Surgery/General Thoracic/Pulmonary Surgery/Esophageal Surgery  2: Nurse Practitioner*/Special*, Nurs*/Nurs* Special*/Nurs* Personnel*/Professional Nurse/Advanced Practice Nurse/Nurs*  3: Curriculum/education/Curricula/Short Term Course*/Course*, Short-Term/training/cultivate/teaching  4=1 AND 2AND 3 | 932 |
| CNKI | 主题：胸外科 + 心胸外科 + 胸心外科 + 普胸外科 + 普胸 + 肺外科 + 食管外科）AND（主题：专科护士 + 护理专家 + 专业护士 + 高级实践护士 + 执业护士 + 护理）AND（主题：课程 + 培训 + 培养 + 方案 + 体系 | 276 |
| Wanfang | 主题:(胸外科 OR 心胸外科 OR 胸心外科 OR 普胸外科 OR 普胸 OR 肺外科 OR 食管外科) and 主题:(专科护士 OR 护理专家 OR 专业护士 OR 高级实践护士 OR 执业护士 OR 护理) and 主题:(课程 OR 培训 OR 培养 OR 方案 OR 体系) | 1475 |
| VIP | ((((((((题名或关键词=胸外科 OR 题名或关键词=心胸外科) OR 题名或关键词=胸心外科) OR 题名或关键词=普胸外科) OR 题名或关键词=普胸) OR 题名或关键词=肺外科) OR 题名或关键词=食管外科) AND (((((题名或关键词=专科护士 OR 题名或关键词=护理专家) OR 题名或关键词=专业护士) OR 题名或关键词=高级实践护士) OR 题名或关键词=执业护士) OR 题名或关键词=护理)) AND ((((题名或关键词=课程 OR 题名或关键词=培训) OR 题名或关键词=培养) OR 题名或关键词=方案) OR 题名或关键词=体系)) | 71 |
| CMB | ( "胸外科"[常用字段:智能] OR "心胸外科"[常用字段:智能] OR "胸心外科"[常用字段:智能] OR "普胸外科"[常用字段:智能] OR "普胸"[常用字段:智能] OR "肺外科"[常用字段:智能] OR "食管外科"[常用字段:智能]) AND( "专科护士"[常用字段:智能] OR "护理专家"[常用字段:智能] OR "专业护士"[常用字段:智能] OR "高级实践护士"[常用字段:智能] OR "执业护士"[常用字段:智能] OR "护理"[常用字段:智能]) AND( "课程"[常用字段:智能] OR "培训"[常用字段:智能] OR "培养"[常用字段:智能] OR "方案"[常用字段:智能] OR "体系"[常用字段:智能]) | 620 |
| Total |  | 6354 |
