# Supplementary material for: Construction of the core competencies training system for thoracic surgery specialist nurses: A mixed-methods study
Source: PLoS One. 2026 Feb 2;21(2):e0339777. doi: 10.1371/journal.pone.0339777 (PMC12863540; doi:10.1371/journal.pone.0339777)
Supplement: S1 File — (ZIP) [file pone.0339777.s001.zip › S3 File. Semi-structured interviews (Informed consent form and demographic questionnaire).docx]

**Semistructured interview informed consent form**

Dear nursing professional/expert,

I sincerely invite you to participate in an semistructured interview regarding the core competency training for thoracic surgery specialized nurses. Please carefully read the following information and consider whether to engage in this interview.

Lung cancer, as one of the primary diseases in thoracic surgery, has consistently high incidence and mortality rates. The large patient population and high postoperative complication rate pose severe challenges to thoracic nursing in China. With the growing health needs of thoracic surgery patients and the development of specialized techniques, thoracic surgery has gradually separated from cardiothoracic surgery to become an independent specialty, responding to the disciplinary development requirements, adapting to specialized business expansion, and addressing the complexity and specialization of various diseases. This separation helps improve professional standards and treatment outcomes in the field but also imposes more refined, specialized, and systematic requirements on the competencies of thoracic surgery specialized nurses.

Currently, thoracic surgery specialized nurses in China are in a developmental stage. This study aims to construct a training program for thoracic surgery specialized nurses suitable for China’s nursing context based on core competency theory. Before developing this program, we hope to understand your perceptions, insights, reflections, and level of knowledge regarding the cultivation of thoracic surgery specialized nurses, with the goal of refining the core competency standards for thoracic surgery specialized nurses and promoting high-quality development of specialized thoracic nursing services.

This interview will be conducted anonymously to ensure your candid sharing of insights, with an estimated duration of 30 to 60 minutes. To facilitate systematic qualitative analysis, the conversation will be audio-recorded. However, all materials will be de-identified and used exclusively for this research purpose. Your personal information and privacy will be strictly protected: no identifiable data will be included in any publications or reports, and all records linked to you will be permanently destroyed upon study completion. You retain the absolute right to withdraw from the interview at any time without providing reasons, and such a decision will have no impact on your professional or personal life. We sincerely appreciate your contribution to advancing evidence-based training for thoracic surgery specialized nurses.

**Informed consent statement:**

I have carefully read this informed consent form, and the researcher has fully explained the study’s objectives, content, and potential risks to me. I understand the nature of this research and, after careful consideration, voluntarily agree to participate in this interview.

Participant’s signature: _________________

Date: _______________________________

**Semi-structured interview demographic questionnaire (Nursing professionals)**

ID: __________ Interview Date: __________ Interview Location: __________

Please fill in the blanks or check the boxes (☐) according to your actual situation.

1. Age: __________ years old
2. Gender: ☐Female ☐Male
3. Marital status: ☐Single ☐Married ☐Divorced ☐Widowed
4. Professional title: ☐Junior professional title ☐Intermediate professional title

☐Deputy senior professional title ☐Senior professional title

1. Duties: ☐Responsible nurse ☐Responsible group leader

☐Nursing management post

1. Highest education level: ______________________________________
2. Working experience in clinical nursing: ___________________ years
3. Working experience in thoracic surgery clinical nursing: __________ years
4. Hospital name: ______________________________________________
5. Current department: _________________________________________
6. Have you received thoracic surgery-related training?

☐Yes: Frequency (times/month): ☐<2 ☐2-4 ☐5-8 ☐>8

☐No: Never received any relevant training

**Semi-structured interview demographic questionnaire (experts)**

ID: __________ Interview Date: __________ Interview Location: __________

Please fill in the blanks or check the boxes (☐) according to your actual situation.

1. Age: __________ years old
2. Gender: ☐Female ☐Male
3. Marital status: ☐Single ☐Married ☐Divorced ☐Widowed
4. Professional title: ☐Junior professional title ☐Intermediate professional title

☐Deputy senior professional title ☐Senior professional title

1. Duties: ______________________________________________________
2. Highest educational degree:

☐Bachelor’s degree ☐Master’s degree ☐ Doctoral degree

1. Work domains (multiple selections allowed):

☐Thoracic surgery clinical nursing ☐Nursing management

☐Thoracic surgery clinical medicine ☐Other: ____________

1. Working experience in clinical nursing: ___________________ years
2. Working experience in thoracic surgery clinical nursing: __________ years
