# Supplementary material for: Construction of the core competencies training system for thoracic surgery specialist nurses: A mixed-methods study
Source: PLoS One. 2026 Feb 2;21(2):e0339777. doi: 10.1371/journal.pone.0339777 (PMC12863540; doi:10.1371/journal.pone.0339777)
Supplement: S1 File — (ZIP) [file pone.0339777.s001.zip › S4 File. Delphi round 1-The expert consultation questionnaire.docx]

**Delphi round 1: The expert consultation questionnaire**

**Construction of the core competencies training system for thoracic surgery specialist nurses:**

**A mixed-methods study**

Dear Expert,

With the deepened specialization of thoracic surgery, the growing health needs of thoracic patients, and the advancement of specialized techniques, the high volume of surgeries, complex procedures, and high postoperative complication rates pose severe challenges to thoracic nursing in China. These demands also require thoracic surgery nurses to possess more refined, specialized, and systematic core competencies. Studies indicate that specialist nurses significantly improve patient outcomes, including health status, functional capacity, and quality of life, while reducing hospitalization duration, mortality, readmission rates, and healthcare costs, contributing to national healthcare sustainability and high-quality nursing service development. However, thoracic surgery nursing in China has a relatively short history, with thoracic surgery specialist nurses training still in its infancy. Only select regions have begun exploring standalone training programs, and a comprehensive national framework remains undeveloped. **Therefore, this study aims to construct a core competencies training system for thoracic surgery specialist nurses.**

Guided by the Core Competency Theory, this research seeks to **define the core competency framework for thoracic surgery specialist nurses**. The training system will be structured around competency requirements, covering **a core competency framework (training objectives), training curriculum contents along with their corresponding teaching methods, organizational management, and evaluation methods.** A preliminary training system has been developed through literature review and qualitative interviews. In this phase, we employ the Delphi method to finalize the indicators. After each round of consultation, the research team will synthesize expert feedback to refine the framework until consensus is reached. We now seek your input on the rationality and clarity of indicator names and content.

Given your extensive clinical experience and academic expertise in thoracic surgery clinical nursing, nursing education, and nursing management, we respectfully invite you to serve as an expert in this study. The questionnaire comprises three sections. Please provide your feedback for each item. To ensure research timeliness, kindly return the completed questionnaire via email within **two weeks**. All information will be kept strictly confidential.

Your insights will serve as critical foundations for this research. We sincerely appreciate your guidance and support! Wishing you good health and professional success.

Contact person: Li Yingjin

Email: lyjinemail@163.com

**Questionnaire directory:**

Section 1: Basic information of experts

Section 2: The preliminary core competencies training system for thoracic surgery specialist nurses

1. Core competency framework (training objectives) for thoracic surgery specialist nurses
2. Training curriculum contents along with their corresponding teaching methods for thoracic surgery specialist nurses

(3) Organizational management for thoracic surgery specialist nurses

(4) Evaluation methods for thoracic surgery specialist nurses

Section 3: Expert familiarity and judgement with the content of the survey

**Instructions for completion:**

1. Importance rating: Please rate the importance of each indicator using the Likert scale (5=Very Important, 4=Important, 3=Average, 2=Unimportant, 1=Very Unimportant) by checking the corresponding "☐" in the "Importance Score" column.
2. Modification/Deletion suggestions: If an item requires revision or deletion, please specify your feedback in the "Modification Comments" column.
3. Additional items: If you identify missing items at this level, please list them in the "Items to Add" column.

**Section 1: Basic information of experts**

This section is solely for analyzing the overall profile of consulting experts in this study and adheres strictly to confidentiality principles. Please provide your personal information by checking the appropriate boxes or filling in the blanks. Additional notes may be added in the corresponding columns as needed.

1. Name: __________________
2. Age: ____________________
3. Gender: □Female □Male
4. Highest academic degree: □Bachelor’s degree □Master’s degree □Doctoral degree
5. Professional domain: □Clinical nursing □Clinical medicine □Nursing management □Nursing education □Other: ___________
6. Position: □Head nurse □Deputy director/Director of nursing department □Other: _______________________________________
7. Professional title: □Deputy senior professional title ☐Senior professional title □Other: ____________________________________
8. Contact information: _________________________
9. Email: _____________________________________
10. Hospital name: _____________________________
11. Professional experience: _________________ years

**Section 2: The preliminary core competencies training system for thoracic surgery specialist nurses**

**Table 1 First-level indicators of core competency framework (training objectives) for thoracic surgery specialist nurses**

Please review the following first-level indicators and their definitions. If you agree with the establishment of the indicator and its connotation, kindly place a tick “√” in the corresponding “Importance Rating” column. If modifications are required, please revise the content in the “Modification Suggestions” column. Additional items may be added in the space provided below.

| **First-level indicators** | **Importance rating** | | | | | **Modification suggestions** |
| --- | --- | --- | --- | --- | --- | --- |
|  | **5** | **4** | **3** | **2** | **1** |  |
| Ⅰ Practical competency in thoracic surgical nursing | □ | □ | □ | □ | □ |  |
| Ⅱ Guidance and training competency | □ | □ | □ | □ | □ |  |
| Ⅲ Communication and coordination competency | □ | □ | □ | □ | □ |  |
| Ⅳ Scientific research and innovation competency | □ | □ | □ | □ | □ |  |
| Ⅴ Management competency | □ | □ | □ | □ | □ |  |
| Ⅵ Professional ethics and judgment competency | □ | □ | □ | □ | □ |  |
| **Indicators to be added:** | | | | | | |

If you have any other suggestions, please specify: ____________________________________________________________________________

**Table 2 Second-level indicators of core competency framework (training objectives) for thoracic surgery specialist nurses**

This section developed second-level indicators based on the results of first-level indicators to construct a core competency framework (by which first-level indicators are further guided and interpreted through second-level indicators). Specifically, if you agree with the establishment of the following “second-level indicators,” please check “√” in the corresponding “Importance rating” column. If modification is deemed necessary, please revise the content in the “Modification Suggestions” column. For additional items, please add them in the space provided below.

| **First-level indicators** | **Second-level indicators** | **Importance rating** | | | | | **Modification suggestions** |
| --- | --- | --- | --- | --- | --- | --- | --- |
|  |  | **5** | **4** | **3** | **2** | **1** |  |
| Ⅰ Practical competency in thoracic surgical nursing | Ⅰ-1 Proficiency in professional theoretical knowledge | □ | □ | □ | □ | □ |  |
|  | Ⅰ-2 Specialized technical and practical competency | □ | □ | □ | □ | □ |  |
|  | Ⅰ-3 Nursing assessment competency | □ | □ | □ | □ | □ |  |
|  | Ⅰ-4 Emergency and critical care competency | □ | □ | □ | □ | □ |  |
|  | Ⅰ-5 Critical thinking competency | □ | □ | □ | □ | □ |  |
|  | Ⅰ-6 Application competency of related professional knowledge | □ | □ | □ | □ | □ |  |
|  | **Second-level indicators to be added:** | | | | | | |
| Ⅱ Guidance and training competency | Ⅱ-1 Health education competency | □ | □ | □ | □ | □ |  |
|  | Ⅱ-2 Teaching and training competency | □ | □ | □ | □ | □ |  |
|  | **Second-level indicators to be added:** | | | | | | |

| Ⅲ Communication and coordination competency | Ⅲ-1 Communication competency | □ | □ | □ | □ | □ |  |
| --- | --- | --- | --- | --- | --- | --- | --- |
|  | Ⅲ-2 Collaboration competency | □ | □ | □ | □ | □ |  |
|  | **Second-level indicators to be added:** | | | | | | |
| Ⅳ Scientific research and innovation competency | Ⅳ-1 Self-directed learning competency | □ | □ | □ | □ | □ |  |
|  | Ⅳ-2 Basic scientific research competency | □ | □ | □ | □ | □ |  |
|  | Ⅳ-3 Research translation competency | □ | □ | □ | □ | □ |  |
|  | **Second-level indicators to be added:** | | | | | | |
| Ⅴ Management competency | Ⅴ-1 Planning competency | □ | □ | □ | □ | □ |  |
|  | Ⅴ-2 Organizational competency | □ | □ | □ | □ | □ |  |
|  | Ⅴ-3 Leadership competency | □ | □ | □ | □ | □ |  |
|  | Ⅴ-4 Control Competency | □ | □ | □ | □ | □ |  |
|  | **Second-level indicators to be added:** | | | | | | |
| Ⅵ Professional ethics and judgment competency | Ⅵ-1 Ethical decision-making competency | □ | □ | □ | □ | □ |  |
|  | Ⅵ-2 Individual traits | □ | □ | □ | □ | □ |  |
|  | **Second-level indicators to be added:** | | | | | | |

If you have any other suggestions, please specify: ____________________________________________________________________________

**Table 3 Training curriculum contents along with their corresponding teaching methods for thoracic surgery specialist nurses**

Training curriculum contents were listed according to the first-and second-level indicators of the core competency framework. If you agree with the establishment of the following “training curriculum contents,” please check “√” in the corresponding “Importance Rating” column. Please select appropriate teaching method by checking “√” in the “Teaching methods” column in conjunction with each training curriculum content, where each content may correspond to one, two, or multiple teaching methods. If modifications are required, please revise them in the “Modification Suggestions” column; for additional items, please add them in the space provided below.

The specific teaching forms are explained as follows:

①Lecture-based theoretical teaching

②Skills-oriented training methodology

③ Scenario simulation pedagogy

④Experiential sharing approach

⑤Case-driven discussion framework

⑥Workshop-based interactive learning

⑦Literature-driven research methodology

| **First-level indicators** | **Second-level indicators** | **Training curriculum contents** | **Importance rating** | | | | | **Teaching methods** | | | | | | | **Modification suggestions** |
| --- | --- | --- | --- | --- | --- | --- | --- | --- | --- | --- | --- | --- | --- | --- | --- |
|  |  |  | **5** | **4** | **3** | **2** | **1** | **①** | **②** | **③** | **④** | **⑤** | **⑥** | **⑦** |  |
| Ⅰ Practical competency in thoracic surgical nursing | Ⅰ-1 Proficiency in professional theoretical knowledge | Ⅰ-1-1 Regional thoracic anatomy and physiology | □ | □ | □ | □ | □ | □ | □ | □ | □ | □ | □ | □ |  |
|  |  | Ⅰ-1-2 General diagnostic and therapeutic principles and nursing routine for thoracic diseases | □ | □ | □ | □ | □ | □ | □ | □ | □ | □ | □ | □ |  |
|  |  | Ⅰ-1-3 Basic knowledge of electrocardiogram and identification of common arrhythmias | □ | □ | □ | □ | □ | □ | □ | □ | □ | □ | □ | □ |  |
|  |  | **Third-level indicators to be added:** | | | | | | | | | | | | | |
|  | Ⅰ-2 Specialized technical and practical competency | Ⅰ-2-1 Indications and operation protocols for specialized thoracic surgical equipment (mechanical expectoration devices, respiratory trainers, respiratory humidification therapy devices, red light therapeutic apparatus, enteral nutrition pumps, etc.) | □ | □ | □ | □ | □ | □ | □ | □ | □ | □ | □ | □ |  |
|  |  | Ⅰ-2-2 Management and nursing of patients with pulmonary diseases (lung cancer, pulmonary nodules, pulmonary bullae, etc.) | □ | □ | □ | □ | □ | □ | □ | □ | □ | □ | □ | □ |  |
|  |  | Ⅰ-2-3 Management and nursing of patients with esophageal diseases (esophageal cancer, esophageal leiomyoma, esophageal stromal tumor, etc.) | □ | □ | □ | □ | □ | □ | □ | □ | □ | □ | □ | □ |  |
|  |  | Ⅰ-2-4 Management and nursing of patients with tracheal diseases (tracheal tumors, tracheal foreign bodies, etc.) | □ | □ | □ | □ | □ | □ | □ | □ | □ | □ | □ | □ |  |
|  |  | Ⅰ-2-5 Management and nursing of patients with mediastinal diseases (thymoma, mediastinal emphysema, etc.) | □ | □ | □ | □ | □ | □ | □ | □ | □ | □ | □ | □ |  |
|  |  | Ⅰ-2-6 Management and nursing of patients with chest wall diseases (pectus excavatum, pectus carinatum, chest wall tumors, etc.) | □ | □ | □ | □ | □ | □ | □ | □ | □ | □ | □ | □ |  |
|  |  | Ⅰ-2-7 Management and nursing of patients with thoracic trauma (rib fractures, hemopneumothorax, pulmonary contusion, etc.) | □ | □ | □ | □ | □ | □ | □ | □ | □ | □ | □ | □ |  |
|  |  | Ⅰ-2-8 Management and nursing of patients with other thoracic surgical conditions (hyperhidrosis, empyema, etc.) | □ | □ | □ | □ | □ | □ | □ | □ | □ | □ | □ | □ |  |
|  |  | Ⅰ-2-9 Operation coordination and postoperative care of common thoracic surgical diseases | □ | □ | □ | □ | □ | □ | □ | □ | □ | □ | □ | □ |  |
|  |  | Ⅰ-2-10 Operation coordination and postoperative care of lung transplantation | □ | □ | □ | □ | □ | □ | □ | □ | □ | □ | □ | □ |  |
|  |  | Ⅰ-2-11 Preoperative auxiliary localization techniques for pulmonary nodules and postoperative care | □ | □ | □ | □ | □ | □ | □ | □ | □ | □ | □ | □ |  |
|  |  | Ⅰ-2-12 Operation cooperation and postoperative care of interventional therapy for thoracic tumor patients | □ | □ | □ | □ | □ | □ | □ | □ | □ | □ | □ | □ |  |
|  |  | Ⅰ-2-13 Operative coordination and postoperative nursing for thoracentesis | □ | □ | □ | □ | □ | □ | □ | □ | □ | □ | □ | □ |  |
|  |  | Ⅰ-2-14 Operative coordination and nursing for bedside fiberoptic bronchoscopic suctioning | □ | □ | □ | □ | □ | □ | □ | □ | □ | □ | □ | □ |  |
|  |  | Ⅰ-2-15 Perioperative complications management for common thoracic surgical conditions (pulmonary infection, atelectasis, hemorrhage, persistent pulmonary air leak, bronchopleural fistula, chylothorax, esophageal anastomotic fistula, esophagotracheal fistula, venous thromboembolism, arrhythmia, etc.) | □ | □ | □ | □ | □ | □ | □ | □ | □ | □ | □ | □ |  |
|  |  | Ⅰ-2-16 Nursing of thoracic surgical catheters (chest closed drainage tubes, mediastinal drainage tubes, gastric tubes, nasoenteric tubes, jejunostomy tubes, central venous catheters, PICC, etc.) | □ | □ | □ | □ | □ | □ | □ | □ | □ | □ | □ | □ |  |
|  |  | Ⅰ-2-17 Perioperative pain management for thoracic surgical conditions | □ | □ | □ | □ | □ | □ | □ | □ | □ | □ | □ | □ |  |
|  |  | Ⅰ-2-18 Perioperative airway management and pulmonary rehabilitation strategies for thoracic diseases | □ | □ | □ | □ | □ | □ | □ | □ | □ | □ | □ | □ |  |
|  |  | Ⅰ-2-19 Perioperative nutritional management for thoracic surgical | □ | □ | □ | □ | □ | □ | □ | □ | □ | □ | □ | □ |  |
|  |  | Ⅰ-2-20 Prevention and nursing of postoperative nausea and vomiting in thoracic surgery | □ | □ | □ | □ | □ | □ | □ | □ | □ | □ | □ | □ |  |
|  |  | Ⅰ-2-21 Perioperative blood glucose management for thoracic surgical conditions | □ | □ | □ | □ | □ | □ | □ | □ | □ | □ | □ | □ |  |
|  |  | Ⅰ-2-22 Enhanced recovery after surgery (ERAS) in thoracic surgery from a nursing perspective | □ | □ | □ | □ | □ | □ | □ | □ | □ | □ | □ | □ |  |
|  |  | Ⅰ-2-23 Daytime ward management processes and applications for thoracic surgical procedures | □ | □ | □ | □ | □ | □ | □ | □ | □ | □ | □ | □ |  |
|  |  | Ⅰ-2-24 Nursing care for thoracic surgical patients with comorbid chronic diseases (hypertension, diabetes mellitus, COPD, etc.) | □ | □ | □ | □ | □ | □ | □ | □ | □ | □ | □ | □ |  |
|  |  | **Third-level indicators to be added:** | | | | | | | | | | | | | |
|  | Ⅰ-3 Nursing assessment competency | Ⅰ-3-1 Commonly used perioperative assessment tools in thoracic surgery | □ | □ | □ | □ | □ | □ | □ | □ | □ | □ | □ | □ |  |
|  |  | Ⅰ-3-2 Clinical assessment of thoracic diseases (including laboratory test indicators) and adjunctive examinations | □ | □ | □ | □ | □ | □ | □ | □ | □ | □ | □ | □ |  |
|  |  | Ⅰ-3-3 Chest X-ray interpretation | □ | □ | □ | □ | □ | □ | □ | □ | □ | □ | □ | □ |  |
|  |  | Ⅰ-3-4 Lung auscultation | □ | □ | □ | □ | □ | □ | □ | □ | □ | □ | □ | □ |  |
|  |  | **Third-level indicators to be added:** | | | | | | | | | | | | | |
|  | Ⅰ-4 Emergency and critical care competency | Ⅰ-4-1 Operation of resuscitation equipment (defibrillators, ventilators, bag-valve-mask devices, etc.) | □ | □ | □ | □ | □ | □ | □ | □ | □ | □ | □ | □ |  |
|  |  | Ⅰ-4-2 Interpretation of the latest cardiopulmonary resuscitation (CPR) guidelines and practical training | □ | □ | □ | □ | □ | □ | □ | □ | □ | □ | □ | □ |  |
|  |  | Ⅰ-4-3 Precision fluid management for critically ill thoracic surgical patients | □ | □ | □ | □ | □ | □ | □ | □ | □ | □ | □ | □ |  |
|  |  | Ⅰ-4-4 Recognition and emergency nursing for patients with thoracic massive hemorrhage | □ | □ | □ | □ | □ | □ | □ | □ | □ | □ | □ | □ |  |
|  |  | Ⅰ-4-5 Blood transfusion procedures and management in thoracic surgery | □ | □ | □ | □ | □ | □ | □ | □ | □ | □ | □ | □ |  |
|  |  | Ⅰ-4-6 Recognition and emergency nursing for pulmonary embolism patients | □ | □ | □ | □ | □ | □ | □ | □ | □ | □ | □ | □ |  |
|  |  | Ⅰ-4-7 Recognition and emergency nursing for cerebral infarction patients | □ | □ | □ | □ | □ | □ | □ | □ | □ | □ | □ | □ |  |
|  |  | Ⅰ-4-8 Recognition and emergency nursing for myasthenic crisis in thoracic surgical patients | □ | □ | □ | □ | □ | □ | □ | □ | □ | □ | □ | □ |  |
|  |  | Ⅰ-4-9 Recognition and emergency nursing for postoperative diabetic ketoacidosis in thoracic surgical patients | □ | □ | □ | □ | □ | □ | □ | □ | □ | □ | □ | □ |  |
|  |  | Ⅰ-4-10 Emergency management of thoracic drainage tube dislodgement | □ | □ | □ | □ | □ | □ | □ | □ | □ | □ | □ | □ |  |
|  |  | Ⅰ-4-12 Key points and nursing care for transferring critically ill thoracic surgical patients | □ | □ | □ | □ | □ | □ | □ | □ | □ | □ | □ | □ |  |
|  |  | Ⅰ-4-13 Use and management of emergency medications, psychotropic/narcotic drugs, and high-alert medications | □ | □ | □ | □ | □ | □ | □ | □ | □ | □ | □ | □ |  |
|  |  | **Third-level indicators to be added:** | | | | | | | | | | | | | |
|  | Ⅰ-5 Critical thinking competency | Ⅰ-5-1 Application of evidence-based nursing in thoracic surgical care | □ | □ | □ | □ | □ | □ | □ | □ | □ | □ | □ | □ |  |
|  |  | Ⅰ-5-2 Cultivation of critical thinking competency in thoracic nursing practice | □ | □ | □ | □ | □ | □ | □ | □ | □ | □ | □ | □ |  |
|  |  | Ⅰ-5-2 Application and value of critical thinking competency in thoracic nursing practice | □ | □ | □ | □ | □ | □ | □ | □ | □ | □ | □ | □ |  |
|  |  | **Third-level indicators to be added:** | | | | | | | | | | | | | |
|  | Ⅰ-6 Application competency of related professional knowledge | Ⅰ-6-1 Palliative care for patients with advanced thoracic malignancies | □ | □ | □ | □ | □ | □ | □ | □ | □ | □ | □ | □ |  |
|  |  | Ⅰ-6-2 Application of Traditional Chinese Medicine (TCM) nursing techniques in thoracic surgery care | □ | □ | □ | □ | □ | □ | □ | □ | □ | □ | □ | □ |  |
|  |  | Ⅰ-6-3 Writing nursing documents and conducting tertiary care rounds for critically ill patients | □ | □ | □ | □ | □ | □ | □ | □ | □ | □ | □ | □ |  |
|  |  | Ⅰ-6-4 Nosocomial infection control and occupational risk protection | □ | □ | □ | □ | □ | □ | □ | □ | □ | □ | □ | □ |  |
|  |  | Ⅰ-6-5 Development process and global status of specialized nursing clinics in thoracic surgery | □ | □ | □ | □ | □ | □ | □ | □ | □ | □ | □ | □ |  |
|  |  | Ⅰ-6-6 Reception processes and nursing implementation in thoracic surgical nursing clinics | □ | □ | □ | □ | □ | □ | □ | □ | □ | □ | □ | □ |  |
|  |  | **Third-level indicators to be added:** | | | | | | | | | | | | | |
| Ⅱ Guidance and training competency | Ⅱ-1 Health education competency | Ⅱ-1-1 Individualized health education and discharge guidance for thoracic surgical patients | □ | □ | □ | □ | □ | □ | □ | □ | □ | □ | □ | □ |  |
|  |  | Ⅱ-1-2 Production and dissemination of popularization of science for thoracic surgery care | □ | □ | □ | □ | □ | □ | □ | □ | □ | □ | □ | □ |  |
|  |  | Ⅱ-1-3 Application of continuous care in thoracic surgery | □ | □ | □ | □ | □ | □ | □ | □ | □ | □ | □ | □ |  |
|  |  | Ⅱ-1-4 Self-management guidance for thoracic surgical patients | □ | □ | □ | □ | □ | □ | □ | □ | □ | □ | □ | □ |  |
|  |  | **Third-level indicators to be added:** | | | | | | | | | | | | | |
|  | Ⅱ-2 Teaching and training competency | Ⅱ-2-1 Integration of professional ethics in clinical nursing teaching design | □ | □ | □ | □ | □ | □ | □ | □ | □ | □ | □ | □ |  |
|  |  | Ⅱ-2-2 Methods and techniques for clinical nursing teaching | □ | □ | □ | □ | □ | □ | □ | □ | □ | □ | □ | □ |  |
|  |  | Ⅱ-2-3 Hierarchical management and practice in clinical nursing education | □ | □ | □ | □ | □ | □ | □ | □ | □ | □ | □ | □ |  |
|  |  | **Third-level indicators to be added:** | | | | | | | | | | | | | |
| Ⅲ Communication and coordination competency | Ⅲ-1 Communication competency | Ⅲ-1-1 Communication methods and verbal/nonverbal communication skills | □ | □ | □ | □ | □ | □ | □ | □ | □ | □ | □ | □ |  |
|  |  | Ⅲ-1-2 Recognition and management of medical-nursing disputes | □ | □ | □ | □ | □ | □ | □ | □ | □ | □ | □ | □ |  |
|  |  | **Third-level indicators to be added:** | | | | | | | | | | | | | |
|  | Ⅲ-2 Coordination competency | Ⅲ-2-1 Develop interpersonal skills | □ | □ | □ | □ | □ | □ | □ | □ | □ | □ | □ | □ |  |
|  |  | Ⅲ-2-2 Interdepartmental and multidisciplinary team collaboration protocols and processes | □ | □ | □ | □ | □ | □ | □ | □ | □ | □ | □ | □ |  |
|  |  | Ⅲ-2-3 Principles and procedures for consultation and referral | □ | □ | □ | □ | □ | □ | □ | □ | □ | □ | □ | □ |  |
|  |  | **Third-level indicators to be added:** | | | | | | | | | | | | | |
| Ⅳ Scientific research and innovation competency | Ⅳ-1 Self-directed learning competency | Ⅳ-1-1 Implementation and reflection on the latest consensus guidelines and group standards in thoracic surgery | □ | □ | □ | □ | □ | □ | □ | □ | □ | □ | □ | □ |  |
|  |  | Ⅳ-1-2 Learning methods and strategies in thoracic surgery | □ | □ | □ | □ | □ | □ | □ | □ | □ | □ | □ | □ |  |
|  |  | **Third-level indicators to be added:** | | | | | | | | | | | | | |
|  | Ⅳ-2 Basic scientific research competency | Ⅳ-2-1 Literature retrieval strategies and hands-on practice | □ | □ | □ | □ | □ | □ | □ | □ | □ | □ | □ | □ |  |
|  |  | Ⅳ-2-2 Application of literature management software | □ | □ | □ | □ | □ | □ | □ | □ | □ | □ | □ | □ |  |
|  |  | Ⅳ-2-3 Topic selection and research design in nursing research | □ | □ | □ | □ | □ | □ | □ | □ | □ | □ | □ | □ |  |
|  |  | Ⅳ-2-4 Formatting and standards for research proposal development | □ | □ | □ | □ | □ | □ | □ | □ | □ | □ | □ | □ |  |
|  |  | Ⅳ-2-5 Evidence-based nursing and clinical practice integration | □ | □ | □ | □ | □ | □ | □ | □ | □ | □ | □ | □ |  |
|  |  | Ⅳ-1-7 Fundamentals of medical statistics and software applications | □ | □ | □ | □ | □ | □ | □ | □ | □ | □ | □ | □ |  |
|  |  | Ⅳ-1-8 Standardized writing and submission of nursing research papers | □ | □ | □ | □ | □ | □ | □ | □ | □ | □ | □ | □ |  |
|  |  | **Third-level indicators to be added:** | | | | | | | | | | | | | |
|  | Ⅳ-3 Research translation competency | Ⅳ-3-1 Application and writing of nursing patents | □ | □ | □ | □ | □ | □ | □ | □ | □ | □ | □ | □ |  |
|  |  | Ⅳ-3-2 Cultivation of innovative thinking competency and technological innovation in nursing | □ | □ | □ | □ | □ | □ | □ | □ | □ | □ | □ | □ |  |
|  |  | **Third-level indicators to be added:** | | | | | | | | | | | | | |
| Ⅴ Management competency | Ⅴ-1 Planning competency | Ⅴ-1-1 Development and implementation of thoracic surgical nursing plans | □ | □ | □ | □ | □ | □ | □ | □ | □ | □ | □ | □ |  |
|  |  | Ⅴ-1-2 Whole-patient journey management for thoracic surgical conditions | □ | □ | □ | □ | □ | □ | □ | □ | □ | □ | □ | □ |  |
|  |  | **Third-level indicators to be added:** | | | | | | | | | | | | | |
|  | Ⅴ-2 Organizational competency | Ⅴ-2-1 Formats and execution of clinical nursing rounds in thoracic surgery | □ | □ | □ | □ | □ | □ | □ | □ | □ | □ | □ | □ |  |
|  |  | Ⅴ-2-2 Complex case discussions under the multidisciplinary team (MDT) approach instructional approach | □ | □ | □ | □ | □ | □ | □ | □ | □ | □ | □ | □ |  |
|  |  | Ⅴ-2-3 Nursing workforce allocation and management | □ | □ | □ | □ | □ | □ | □ | □ | □ | □ | □ | □ |  |
|  |  | Ⅴ-2-4 Emergency handling of public health emergencies | □ | □ | □ | □ | □ | □ | □ | □ | □ | □ | □ | □ |  |
|  |  | **Third-level indicators to be added:** | | | | | | | | | | | | | |
|  | Ⅴ-3 Leadership competency | Ⅴ-3-1 Clinical pathway process management in thoracic surgical nursing | □ | □ | □ | □ | □ | □ | □ | □ | □ | □ | □ | □ |  |
|  |  | Ⅴ-3-2 Ward nursing management under the primary nursing model in thoracic surgery | □ | □ | □ | □ | □ | □ | □ | □ | □ | □ | □ | □ |  |
|  |  | Ⅴ-3-3 Development planning for thoracic subspecialty nursing | □ | □ | □ | □ | □ | □ | □ | □ | □ | □ | □ | □ |  |
|  |  | Ⅴ-3-4 Leadership in nursing practice for thoracic surgery | □ | □ | □ | □ | □ | □ | □ | □ | □ | □ | □ | □ |  |
|  |  | **Third-level indicators to be added:** | | | | | | | | | | | | | |
|  | Ⅴ-4 Control Competency | Ⅴ-4-1 Establishment and monitoring of thoracic nursing quality indicators | □ | □ | □ | □ | □ | □ | □ | □ | □ | □ | □ | □ |  |
|  |  | Ⅴ-4-2 Monitoring and management of nursing safety incidents | □ | □ | □ | □ | □ | □ | □ | □ | □ | □ | □ | □ |  |
|  |  | **Third-level indicators to be added:** | | | | | | | | | | | | | |
| Ⅵ Professional ethics and judgment competency | Ⅵ-1 Ethical decision-making competency | Ⅵ-1-1 Laws, regulations and rules related to thoracic surgery | □ | □ | □ | □ | □ | □ | □ | □ | □ | □ | □ | □ |  |
|  |  | Ⅵ-1- 2 Ethics and moral norms of thoracic surgery | □ | □ | □ | □ | □ | □ | □ | □ | □ | □ | □ | □ |  |
|  |  | **Third-level indicators to be added:** | | | | | | | | | | | | | |
|  | Ⅵ-2 Individual traits | Ⅵ-2-1 Clinical practice of humanistic care in thoracic surgery | □ | □ | □ | □ | □ | □ | □ | □ | □ | □ | □ | □ |  |
|  |  | Ⅵ-2-2 Occupational stress and self-regulation for thoracic surgery specialist nurses | □ | □ | □ | □ | □ | □ | □ | □ | □ | □ | □ | □ |  |
|  |  | Ⅵ-2-3 Professional spirit and social responsibility for thoracic surgery specialist nurses | □ | □ | □ | □ | □ | □ | □ | □ | □ | □ | □ | □ |  |
|  |  | Ⅵ-2-4 Professional development and career planning for thoracic surgery specialist nurses | □ | □ | □ | □ | □ | □ | □ | □ | □ | □ | □ | □ |  |
|  |  | **Third-level indicators to be added:** | | | | | | | | | | | | | |

If you have any other suggestions, please specify: ____________________________________________________________________________

**Table 4 Organizational management for thoracic surgery specialist nurses**

| **First-level indicators** | **Second-level indicators** | **Importance rating** | | | | | **Modification suggestions** |
| --- | --- | --- | --- | --- | --- | --- | --- |
|  |  | **5** | **4** | **3** | **2** | **1** |  |
| Ⅰ Training paradigm | Ⅰ-1 Total course hours: 480 hours (45 minutes per hour) | □ | □ | □ | □ | □ |  |
|  | Ⅰ-2 Integrating theoretical knowledge and practical skills | □ | □ | □ | □ | □ |  |
|  | Ⅰ-3 Duration: 3 months, comprising 1 month of theoretical learning and 2 months of practical training | □ | □ | □ | □ | □ |  |
|  | Ⅰ-4 Full-time commitment without work responsibilities | □ | □ | □ | □ | □ |  |
|  | **Indicators to be added:** | | | | | | |
| Ⅱ Admission criteria for trainees | Ⅱ-1 Holding a valid nursing practice qualification certificate | □ | □ | □ | □ | □ |  |
|  | Ⅱ-2 Bachelor’s degree or higher education level | □ | □ | □ | □ | □ |  |
|  | Ⅱ-3 Professional title of Nurse Practitioner or higher | □ | □ | □ | □ | □ |  |
|  | Ⅱ-4 At least 6 years of clinical nursing experience | □ | □ | □ | □ | □ |  |
|  | Ⅱ-5 At least 4 years of nursing experience in thoracic surgery | □ | □ | □ | □ | □ |  |
|  | Ⅱ-6 At least 2 years of clinical nursing teaching experience | □ | □ | □ | □ | □ |  |
|  | Ⅱ-7 Basic English proficiency for academic learning | □ | □ | □ | □ | □ |  |
|  | Ⅱ-8 Demonstrated high ethical standards and professional morality | □ | □ | □ | □ | □ |  |
|  | **Indicators to be added:** | | | | | | |
| Ⅲ Faculty selection criteria | Ⅲ-1 Bachelor’s degree or higher (nurses); master’s degree or higher (physicians) | □ | □ | □ | □ | □ |  |
|  | Ⅲ-2 Intermediate professional title or above (nurses); associate senior or higher professional title (physicians) | □ | □ | □ | □ | □ |  |
|  | Ⅲ-3 At least 10 years of specialized experience in related specialties | □ | □ | □ | □ | □ |  |
|  | Ⅲ-4 At leas 5 years of education experience in academic institutions or clinical training settings | □ | □ | □ | □ | □ |  |
|  | Ⅲ-5 Proven integrity, professional ethics, and dedication to education | □ | □ | □ | □ | □ |  |
|  | **Indicators to be added:** | | | | | | |

If you have any other suggestions, please specify: ____________________________________________________________________________

**Table 5 Evaluation methods for thoracic surgery specialist nurses**

| **First-level indicators** | **Second-level indicators** | **Importance rating** | | | | | **Modification suggestions** |
| --- | --- | --- | --- | --- | --- | --- | --- |
|  |  | **5** | **4** | **3** | **2** | **1** |  |
| Ⅰ Formative evaluation | Ⅰ-1 Class participation | □ | □ | □ | □ | □ |  |
|  | Ⅰ-2 Case analysis | □ | □ | □ | □ | □ |  |
|  | Ⅰ-3 English literature reading presentation | □ | □ | □ | □ | □ |  |
|  | Ⅰ-4 Health education popularization | □ | □ | □ | □ | □ |  |
|  | **Indicators to be added:** | | | | | | |
| Ⅱ Summative evaluation | Ⅱ-1 Specialty theoretical assessment | □ | □ | □ | □ | □ |  |
|  | Ⅱ-2 Specialty skills assessment: Objective Structured Clinical Examination (OSCE) | □ | □ | □ | □ | □ |  |
|  | Ⅱ-3 Case nursing report | □ | □ | □ | □ | □ |  |
|  | Ⅱ-4 Research proposal | □ | □ | □ | □ | □ |  |
|  | **Indicators to be added:** | | | | | | |

If you have any other suggestions, please specify: ____________________________________________________________________________

**Section 3: Expert familiarity and judgement with the content of the survey**

| ****Familiarity level with research questions**** | □Very familiar  □Relatively familiar  □Moderately familiar  □Somewhat unfamiliar  □Not familiar | ****Degree of judgment on research indicators**** | **Basis for judgment** | ****The degree of impact on your judgment**** | | |
| --- | --- | --- | --- | --- | --- | --- |
|  |  |  |  | **High impact** | **Moderate impact** | **Low impact** |
|  |  |  | Theoretical analysis | □ | □ | □ |
|  |  |  | Practical experience | □ | □ | □ |
|  |  |  | Related domestic and international literature/data | □ | □ | □ |
|  |  |  | Subjective judgment | □ | □ | □ |

**The present survey has now reached its conclusion. Once again, we extend our heartfelt gratitude for your invaluable support throughout this process. May your professional journey be filled with continued success and fulfillment.**
