# Supplementary material for: Construction of the core competencies training system for thoracic surgery specialist nurses: A mixed-methods study
Source: PLoS One. 2026 Feb 2;21(2):e0339777. doi: 10.1371/journal.pone.0339777 (PMC12863540; doi:10.1371/journal.pone.0339777)
Supplement: S1 File — (ZIP) [file pone.0339777.s001.zip › S5 File. Delphi round 2-The expert consultation questionnaire.docx]

**Delphi round 2: The expert consultation questionnaire**

**Construction of the core competencies training system for thoracic surgery specialist nurses:**

**A mixed-methods study**

Dear Expert,

With the deepened specialization of thoracic surgery, the growing health needs of thoracic patients, and the advancement of specialized techniques, the high volume of surgeries, complex procedures, and high postoperative complication rates pose severe challenges to thoracic nursing in China. These demands also require thoracic nurses to possess more refined, specialized, and systematic core competencies. Studies indicate that specialist nurses significantly improve patient outcomes, including health status, functional capacity, and quality of life, while reducing hospitalization duration, mortality, readmission rates, and healthcare costs, contributing to national healthcare sustainability and high-quality nursing service development. However, thoracic nursing in China has a relatively short history, with thoracic surgery specialist nurses training still in its infancy. Only select regions have begun exploring standalone training programs, and a comprehensive national framework remains undeveloped. **Therefore, this study aims to construct a core competencies training system for thoracic surgery specialist nurses.**

Guided by the Core Competency Theory, this research seeks to **define the core competency framework for thoracic surgery specialist nurses**. The training system will be structured around competency requirements, covering **a core competency framework (training objectives), training curriculum contents along with their corresponding teaching methods, organizational management, and evaluation methods.**

In the first round of questionnaire consultation, 10 experts put forward their suggestions on the revision of the course system. **After the group discussion, we modified the training system by revising 3 items of the core competency framework, deleting 2 items, revising 19 curriculum contents, adding 6 new curriculum contents, deleting 6 existing curriculum contents, revising 2 organizational management aspects, and revising 2 evaluation methods.** **Revised sections have been marked in red**. We hereby submit the second-round Delphi questionnaire for your review and solicit your feedback regarding the rationality of the indicator names and content. To ensure the study's progress, we kindly request that you send back your comments via Email within two weeks. The research team will strictly safeguard the confidentiality of your data.

Your insights will serve as critical foundations for this research. We sincerely appreciate your guidance and support! Wishing you good health and professional success.

Contact person: Li Yingjin

Email: lyjinemail@163.com

**Questionnaire directory:**

Section 1: Basic information of experts

Section 2: The core competencies course training system for thoracic surgery specialist nurses

1. Core competency framework (training objectives) for thoracic surgery specialist nurses
2. Training curriculum contents along with their corresponding teaching methods for thoracic surgery specialist nurses

(3) Organizational management for thoracic surgery specialist nurses

(4) Evaluation methods for thoracic surgery specialist nurses

Section 3: Expert familiarity and judgement with the content of the survey

**Instructions for completion:**

1. Importance rating: Please rate the importance of each indicator using the Likert scale (5=Very Important, 4=Important, 3=Average, 2=Unimportant, 1=Very Unimportant) by checking the corresponding "☐" in the "Importance Score" column.
2. Modification/Deletion suggestions: If an item requires revision or deletion, please specify your feedback in the "Modification Comments" column.
3. Additional items: If you identify missing items at this level, please list them in the "Items to Add" column.

**Section 1: Basic information of experts**

This section is solely for analyzing the overall profile of consulting experts in this study and adheres strictly to confidentiality principles. Please provide your personal information by checking the appropriate boxes or filling in the blanks. Additional notes may be added in the corresponding columns as needed.

1. Name: __________________
2. Age: ____________________
3. Gender: □Female □Male
4. Highest academic degree: □Bachelor’s degree □Master’s degree □Doctoral degree
5. Professional domain: □Clinical nursing □Clinical medicine □Nursing management □Nursing education □Other: ___________
6. Position: □Head nurse □Deputy director/Director of nursing department □Other: _______________________________________
7. Professional title: □Deputy senior professional title ☐Senior professional title □Other: ____________________________________
8. Contact information: _________________________
9. Email: _____________________________________
10. Hospital name: _____________________________
11. Professional experience: _________________ years

**Section 2: The core competencies training system for thoracic surgery specialist nurses**

**Table 1 First-level indicators of core competency framework (training objectives) for thoracic surgery specialist nurses**

Please review the following first-level indicators and their definitions. If you agree with the establishment of the indicator and its connotation, kindly place a tick “√” in the corresponding “Importance Rating” column. If modifications are required, please revise the content in the “Modification Suggestions” column. Additional items may be added in the space provided below.

| **First-level indicators** | **Importance rating** | | | | | **Modification suggestions** |
| --- | --- | --- | --- | --- | --- | --- |
|  | **5** | **4** | **3** | **2** | **1** |  |
| Ⅰ Practical competency in thoracic surgical nursing | □ | □ | □ | □ | □ |  |
| Ⅱ Guidance and training competency | □ | □ | □ | □ | □ |  |
| Ⅲ Communication and coordination competency | □ | □ | □ | □ | □ |  |
| **Ⅳ Innovative practice competency** | □ | □ | □ | □ | □ |  |
| Ⅴ Management competency | □ | □ | □ | □ | □ |  |
| Ⅵ Professional ethics and judgment competency | □ | □ | □ | □ | □ |  |
| **Indicators to be added:** | | | | | | |

If you have any other suggestions, please specify: ____________________________________________________________________________

**Table 2 Second-level indicators of core competency framework (training objectives) for thoracic surgery specialist nurses**

This section developed second-level indicators based on the results of first-level indicators to construct a core competency framework (by which first-level indicators are further guided and interpreted through second-level indicators). Specifically, if you agree with the establishment of the following “second-level indicators,” please check “√” in the corresponding “Importance rating” column. If modification is deemed necessary, please revise the content in the “Modification Suggestions” column. For additional items, please add them in the space provided below.

| **First-level indicators** | **Second-level indicators** | **Importance rating** | | | | | **Modification suggestions** |
| --- | --- | --- | --- | --- | --- | --- | --- |
|  |  | **5** | **4** | **3** | **2** | **1** |  |
| Ⅰ Practical competency in thoracic surgical nursing | Ⅰ-1 Proficiency in professional theoretical knowledge | □ | □ | □ | □ | □ |  |
|  | Ⅰ-2 Specialized technical and practical competency | □ | □ | □ | □ | □ |  |
|  | Ⅰ-3 Nursing assessment competency | □ | □ | □ | □ | □ |  |
|  | Ⅰ-4 Emergency and critical care competency | □ | □ | □ | □ | □ |  |
|  | Ⅰ-5 Critical thinking competency | □ | □ | □ | □ | □ |  |
|  | **Second-level indicators to be added:** | | | | | | |
| Ⅱ Guidance and training competency | Ⅱ-1 Health education competency | □ | □ | □ | □ | □ |  |
|  | Ⅱ-2 Teaching and training competency | □ | □ | □ | □ | □ |  |
|  | **Second-level indicators to be added:** | | | | | | |
| Ⅲ Communication and coordination competency | Ⅲ-1 Communication competency | □ | □ | □ | □ | □ |  |
|  | **Ⅲ-2 Coordination competency** | □ | □ | □ | □ | □ |  |
|  | **Second-level indicators to be added:** | | | | | | |
| **Ⅳ Innovative practice competency** | **Ⅳ-1 Research and innovation competency** | □ | □ | □ | □ | □ |  |
|  | **Ⅳ-2 Practice translation competency** | □ | □ | □ | □ | □ |  |
|  | **Second-level indicators to be added:** | | | | | | |
| Ⅴ Management competency | Ⅴ-1 Planning competency | □ | □ | □ | □ | □ |  |
|  | Ⅴ-2 Organizational competency | □ | □ | □ | □ | □ |  |
|  | Ⅴ-3 Leadership competency | □ | □ | □ | □ | □ |  |
|  | Ⅴ-4 Control Competency | □ | □ | □ | □ | □ |  |
|  | **Second-level indicators to be added:** | | | | | | |
| Ⅵ Professional ethics and judgment competency | Ⅵ-1 Ethical decision-making competency | □ | □ | □ | □ | □ |  |
|  | **Ⅵ-2 Professional literacy** | □ | □ | □ | □ | □ |  |
|  | **Second-level indicators to be added:** | | | | | | |

If you have any other suggestions, please specify: ____________________________________________________________________________

**Table 3 Training curriculum contents along with their corresponding teaching methods for thoracic surgery specialist nurses**

Training curriculum contents were listed according to the first-and second-level indicators of the core competency framework. If you agree with the establishment of the following “training curriculum contents,” please check “√” in the corresponding “Importance Rating” column. Please select appropriate teaching method by checking “√” in the “Teaching methods” column in conjunction with each training curriculum content, where each content may correspond to one, two, or multiple teaching methods. If modifications are required, please revise them in the “Modification Suggestions” column; for additional items, please add them in the space provided below.

The specific teaching forms are explained as follows:

①Lecture-based theoretical teaching

②Skills-oriented training methodology

③ Scenario simulation pedagogy

④Experiential sharing approach

⑤Case-driven discussion framework

⑥Workshop-based interactive learning

⑦Literature-driven research methodology

| **First-level indicators** | **Second-level indicators** | **Training curriculum contents** | **Teaching**  **methods** | **Importance rating** | | | | | **Modification suggestions** |
| --- | --- | --- | --- | --- | --- | --- | --- | --- | --- |
|  |  |  |  | **5** | **4** | **3** | **2** | **1** |  |
| Ⅰ Practical competency in thoracic surgical nursing | Ⅰ-1 Proficiency in professional theoretical knowledge | Ⅰ-1-1 Regional thoracic anatomy and physiology | ① | □ | □ | □ | □ | □ |  |
|  |  | Ⅰ-1-2 **General diagnostic and therapeutic principles** for thoracic diseases | ① | □ | □ | □ | □ | □ |  |
|  |  | **Ⅰ-1-3 Perioperative nursing protocols for thoracic surgical conditions** | ① | □ | □ | □ | □ | □ |  |
|  |  | **Ⅰ-1-4 Specialized pharmacological knowledge and administration standards in thoracic surgery** | ① | □ | □ | □ | □ | □ |  |
|  |  | **Ⅰ-1-5 Specialized knowledge of thoracic surgical examinations and result interpretation** | ① | □ | □ | □ | □ | □ |  |
|  |  | **Third-level indicators to be added:** | | | | | | | |
|  | Ⅰ-2 Specialized technical and practical competency | Ⅰ-2-1 Indications and operation protocols for specialized thoracic surgical equipment (mechanical expectoration devices, respiratory trainers, respiratory humidification therapy devices, red light therapeutic apparatus, enteral nutrition pumps, etc.) | ② | □ | □ | □ | □ | □ |  |
|  |  | Ⅰ-2-2 Perioperative complications management for common thoracic surgical conditions (pulmonary infection, atelectasis, hemorrhage, persistent pulmonary air leak, bronchopleural fistula, chylothorax, esophageal anastomotic fistula, esophagotracheal fistula, venous thromboembolism, arrhythmia, etc.) | ②④⑤ | □ | □ | □ | □ | □ |  |
|  |  | Ⅰ-2-3 Management and nursing of patients with pulmonary diseases (lung cancer, pulmonary nodules, pulmonary bullae, etc.) | ②⑤ | □ | □ | □ | □ | □ |  |
|  |  | Ⅰ-2-4 Management and nursing of patients with esophageal diseases (esophageal cancer, esophageal leiomyoma, esophageal stromal tumor, etc.) | ②⑤ | □ | □ | □ | □ | □ |  |
|  |  | Ⅰ-2-5 Management and nursing of patients with tracheal diseases (tracheal tumors, tracheal foreign bodies, etc.) | ②⑤ | □ | □ | □ | □ | □ |  |
|  |  | Ⅰ-2-6 Management and nursing of patients with mediastinal diseases (thymoma, mediastinal emphysema, etc.) | ②⑤ | □ | □ | □ | □ | □ |  |
|  |  | Ⅰ-2-7 Management and nursing of patients with chest wall diseases (pectus excavatum, pectus carinatum, chest wall tumors, etc.) | ②⑤ | □ | □ | □ | □ | □ |  |
|  |  | Ⅰ-2-8 Management and nursing of patients with thoracic trauma (rib fractures, hemopneumothorax, pulmonary contusion, etc.) | ②⑤ | □ | □ | □ | □ | □ |  |
|  |  | Ⅰ-2-9 Management and nursing of patients with other thoracic surgical conditions (hyperhidrosis, empyema, etc.) | ②⑤ | □ | □ | □ | □ | □ |  |
|  |  | **Ⅰ-2-10 Common surgical procedures for thoracic diseases and postoperative nursing key points** | ①② | □ | □ | □ | □ | □ |  |
|  |  | **Ⅰ-2-11 Current status and prospects of lung transplantation** | ① | □ | □ | □ | □ | □ |  |
|  |  | Ⅰ-2-12 Development of preoperative auxiliary localization techniques for pulmonary subsolid nodules and related nursing | ①② | □ | □ | □ | □ | □ |  |
|  |  | **Ⅰ-2-13 Advances in interventional therapy for thoracic tumor patients and nursing practice** | ①② | □ | □ | □ | □ | □ |  |
|  |  | Ⅰ-2-14 Operative coordination and postoperative nursing for thoracentesis | ①② | □ | □ | □ | □ | □ |  |
|  |  | Ⅰ-2-15 Operative coordination and nursing for bedside fiberoptic bronchoscopic suctioning | ①② | □ | □ | □ | □ | □ |  |
|  |  | **Ⅰ-2-16 Development of electromagnetic navigation bronchoscopy technology and nursing implications** | ① | □ | □ | □ | □ | □ |  |
|  |  | **Ⅰ-2-17 Current status and prospects of neoadjuvant immunotherapy for non-small cell lung cancer** | ① | □ | □ | □ | □ | □ |  |
|  |  | Ⅰ-2-18 Nursing of thoracic surgical catheters (chest closed drainage tubes, mediastinal drainage tubes, gastric tubes, nasoenteric tubes, jejunostomy tubes, central venous catheters, PICC, etc.) | ①② | □ | □ | □ | □ | □ |  |
|  |  | Ⅰ-2-19 Perioperative pain management for thoracic surgical conditions | ①⑤ | □ | □ | □ | □ | □ |  |
|  |  | Ⅰ-2-20 Perioperative airway management and pulmonary rehabilitation strategies for thoracic diseases | ①④⑤ | □ | □ | □ | □ | □ |  |
|  |  | Ⅰ-2-21 Perioperative nutritional management for thoracic surgical | ①⑤ | □ | □ | □ | □ | □ |  |
|  |  | Ⅰ-2-22 Perioperative blood glucose management for thoracic surgical conditions | ①⑤ | □ | □ | □ | □ | □ |  |
|  |  | Ⅰ-2-23 Enhanced recovery after surgery (ERAS) in thoracic surgery from a nursing perspective | ①⑦ | □ | □ | □ | □ | □ |  |
|  |  | Ⅰ-2-24 Daytime ward management processes and applications for thoracic surgical procedures | ①⑤ | □ | □ | □ | □ | □ |  |
|  |  | Ⅰ-2-25 Nursing care for thoracic surgical patients with comorbid chronic diseases (hypertension, diabetes mellitus, COPD, etc.) | ①④⑤ | □ | □ | □ | □ | □ |  |
|  |  | **Ⅰ-2-26 Continuing nursing care or transitional nursing programs for thoracic surgical conditions** | ① | □ | □ | □ | □ | □ |  |
|  |  | **Ⅰ-2-27 Development process and global status of specialized nursing clinics in thoracic surgery** | ①⑦ | □ | □ | □ | □ | □ |  |
|  |  | **Ⅰ-2-28 Reception processes and nursing implementation in thoracic surgical nursing clinics** | ① | □ | □ | □ | □ | □ |  |
|  |  | **Ⅰ-2-29 Palliative care for patients with advanced thoracic malignancies** | ① | □ | □ | □ | □ | □ |  |
|  |  | **Ⅰ-2-30 Application of Traditional Chinese Medicine (TCM) nursing techniques in thoracic surgery care** | ① | □ | □ | □ | □ | □ |  |
|  |  | **Third-level indicators to be added:** | | | | | | | |

|  | Ⅰ-3 Nursing assessment competency | Ⅰ-3-1 Commonly used perioperative assessment tools in thoracic surgery | ① | □ | □ | □ | □ | □ |  |
| --- | --- | --- | --- | --- | --- | --- | --- | --- | --- |
|  |  | Ⅰ-3-2 Clinical assessment of thoracic diseases (including laboratory test indicators) and adjunctive examinations | ① | □ | □ | □ | □ | □ |  |
|  |  | **Third-level indicators to be added:** | | | | | | | |
|  | Ⅰ-4 Emergency and critical care competency | Ⅰ-4-1 Operation of resuscitation equipment (defibrillators, ventilators, bag-valve-mask devices, etc.) | ②③ | □ | □ | □ | □ | □ |  |
|  |  | Ⅰ-4-2 Precision fluid management for critically ill thoracic surgical patients | ① | □ | □ | □ | □ | □ |  |
|  |  | Ⅰ-4-3 Recognition and emergency nursing for patients with thoracic massive hemorrhage | ①③ | □ | □ | □ | □ | □ |  |
|  |  | Ⅰ-4-4 Blood transfusion procedures and management in thoracic surgery | ①③ | □ | □ | □ | □ | □ |  |
|  |  | Ⅰ-4-5 Recognition and emergency nursing for pulmonary embolism patients | ①③ | □ | □ | □ | □ | □ |  |
|  |  | Ⅰ-4-6 Recognition and emergency nursing for cerebral infarction patients | ①③ | □ | □ | □ | □ | □ |  |
|  |  | Ⅰ-4-7 Recognition and emergency nursing for myasthenic crisis in thoracic surgical patients | ①③ | □ | □ | □ | □ | □ |  |
|  |  | Ⅰ-4-8 Recognition and emergency nursing for postoperative diabetic ketoacidosis in thoracic surgical patients | ①③ | □ | □ | □ | □ | □ |  |
|  |  | Ⅰ-4-9 Emergency management of thoracic drainage tube dislodgement | ③ | □ | □ | □ | □ | □ |  |
|  |  | **Ⅰ-4-10 Recognition and management of common arrhythmias in thoracic surgical patients** | ①⑥ | □ | □ | □ | □ | □ |  |
|  |  | Ⅰ-4-11 Interpretation of the latest cardiopulmonary resuscitation (CPR) guidelines and practical training | ①②⑥ | □ | □ | □ | □ | □ |  |
|  |  | Ⅰ-4-12 Key points and nursing care for transferring critically ill thoracic surgical patients | ①③ | □ | □ | □ | □ | □ |  |
|  |  | Ⅰ-4-13 Use and management of emergency medications, psychotropic/narcotic drugs, and high-alert medications | ① | □ | □ | □ | □ | □ |  |
|  |  | **Ⅰ-4-14 Three-level nursing rounds for critically ill thoracic surgical patients** | ① | □ | □ | □ | □ | □ |  |
|  |  | **Third-level indicators to be added:** | | | | | | | |
|  | Ⅰ-5 Critical thinking competency | Ⅰ-5-1 Application of evidence-based nursing in thoracic surgical care | ①⑦ | □ | □ | □ | □ | □ |  |
|  |  | Ⅰ-5-2 **Cultivation and application** of critical thinking competency in thoracic nursing practice | ①④ | □ | □ | □ | □ | □ |  |
|  |  | **Third-level indicators to be added:** | | | | | | | |
| Ⅱ Guidance and training competency | Ⅱ-1 Health education competency | Ⅱ-1-1 Individualized health education and discharge guidance for thoracic surgical patients | ① | □ | □ | □ | □ | □ |  |
|  |  | Ⅱ-1-2 Production and dissemination of popularization of science for thoracic surgery care | ① | □ | □ | □ | □ | □ |  |
|  |  | Ⅱ-1-3 Self-management guidance for thoracic surgical patients | ① | □ | □ | □ | □ | □ |  |
|  |  | **Third-level indicators to be added:** | | | | | | | |
|  | Ⅱ-2 Teaching and training competency | Ⅱ-2-1 Integration of professional ethics in clinical nursing teaching design | ① | □ | □ | □ | □ | □ |  |
|  |  | Ⅱ-2-2 Methods and techniques for clinical nursing teaching | ①④ | □ | □ | □ | □ | □ |  |
|  |  | Ⅱ-2-3 Hierarchical management and practice in clinical nursing education | ① | □ | □ | □ | □ | □ |  |
|  |  | **Ⅱ-2-4 Curriculum design for thoracic surgical nursing** | ① | □ | □ | □ | □ | □ |  |
|  |  | **Third-level indicators to be added:** | | | | | | | |

| Ⅲ Communication and coordination competency | Ⅲ-1 Communication competency | Ⅲ-1-1 Communication methods and verbal/nonverbal communication skills | ① | □ | □ | □ | □ | □ |  |
| --- | --- | --- | --- | --- | --- | --- | --- | --- | --- |
|  |  | Ⅲ-1-2 Recognition and management of medical-nursing disputes | ① | □ | □ | □ | □ | □ |  |
|  |  | **Third-level indicators to be added:** | | | | | | | |
|  | **Ⅲ-2 Coordination competency** | Ⅲ-2-1 Interdepartmental and multidisciplinary team collaboration protocols and processes | ① | □ | □ | □ | □ | □ |  |
|  |  | **Third-level indicators to be added:** | | | | | | | |
| **Ⅳ Innovative practice competency** | **Ⅳ-1 Research and innovation competency** | Ⅳ-1-1 Implementation and reflection on the latest consensus guidelines and group standards in thoracic surgery | ①⑦ | □ | □ | □ | □ | □ |  |
|  |  | Ⅳ-1-2 Literature retrieval strategies and hands-on practice | ①⑥ | □ | □ | □ | □ | □ |  |
|  |  | Ⅳ-1-3 Application of literature management software | ①⑥ | □ | □ | □ | □ | □ |  |
|  |  | Ⅳ-1-4 Topic selection and research design in nursing research | ①⑦ | □ | □ | □ | □ | □ |  |
|  |  | **Ⅳ-1-5 Topic selection and writing of case nursing reports** | ①⑦ | □ | □ | □ | □ | □ |  |
|  |  | Ⅳ-1-6 Formatting and standards for research proposal development | ① | □ | □ | □ | □ | □ |  |
|  |  | Ⅳ-1-7 Fundamentals of medical statistics and software applications | ① | □ | □ | □ | □ | □ |  |
|  |  | Ⅳ-1-8 Standardized writing and submission of nursing research papers | ① | □ | □ | □ | □ | □ |  |
|  |  | **Third-level indicators to be added:** | | | | | | | |
|  | **Ⅳ-2 Practice translation competency** | **Ⅳ-2-1 Evidence-based nursing and clinical practice integration** | ①⑦ | □ | □ | □ | □ | □ |  |
|  |  | Ⅳ-2-2 Application and **translation** of nursing patents | ①④ | □ | □ | □ | □ | □ |  |
|  |  | **Third-level indicators to be added:** | | | | | | | |
| Ⅴ Management competency | Ⅴ-1 Planning competency | Ⅴ-1-1 Development and implementation of thoracic surgical nursing plans | ① | □ | □ | □ | □ | □ |  |
|  |  | Ⅴ-1-2 Whole-patient journey management for thoracic surgical conditions | ① | □ | □ | □ | □ | □ |  |
|  |  | **Third-level indicators to be added:** | | | | | | | |
|  | Ⅴ-2 Organizational competency | Ⅴ-2-1 Formats and execution of clinical nursing rounds in thoracic surgery | ① | □ | □ | □ | □ | □ |  |
|  |  | Ⅴ-2-2 Complex case discussions under the multidisciplinary team (MDT) approach instructional approach | ①⑤ | □ | □ | □ | □ | □ |  |
|  |  | Ⅴ-2-3 Nursing workforce allocation and management | ① | □ | □ | □ | □ | □ |  |
|  |  | **Ⅴ-2-4 Design and practice of emergency response drills** | ① | □ | □ | □ | □ | □ |  |
|  |  | **Third-level indicators to be added:** | | | | | | | |
|  | Ⅴ-3 Leadership competency | Ⅴ-3-1 Clinical pathway process management in thoracic surgical nursing | ① | □ | □ | □ | □ | □ |  |
|  |  | Ⅴ-3-2 Ward nursing management under the primary nursing model in thoracic surgery | ① | □ | □ | □ | □ | □ |  |
|  |  | Ⅴ-3-3 Development planning for thoracic subspecialty nursing | ① | □ | □ | □ | □ | □ |  |
|  |  | Ⅴ-3-4 Leadership in nursing practice for thoracic surgery | ① | □ | □ | □ | □ | □ |  |
|  |  | **Third-level indicators to be added:** | | | | | | | |
|  | Ⅴ-4 Control Competency | Ⅴ-4-1 Establishment and monitoring of thoracic nursing quality indicators | ① | □ | □ | □ | □ | □ |  |
|  |  | Ⅴ-4-2 Monitoring and management of nursing safety incidents | ① | □ | □ | □ | □ | □ |  |
|  |  | Ⅴ-4-3 Nosocomial infection control and occupational risk protection | ① | □ | □ | □ | □ | □ |  |
|  |  | **Third-level indicators to be added:** | | | | | | | |
| Ⅵ Professional ethics and judgment competency | Ⅵ-1 Ethical decision-making competency | Ⅵ-1-1 **Medical** laws, regulations, and institutional policies | ① | □ | □ | □ | □ | □ |  |
|  |  | Ⅵ-1- 2 **Nursing** ethics and moral standards | ① | □ | □ | □ | □ | □ |  |
|  |  | **Third-level indicators to be added:** | | | | | | | |
|  | **Ⅵ-2 Professional literacy** | Ⅵ-2-1 Clinical practice of humanistic care in thoracic surgery | ① | □ | □ | □ | □ | □ |  |
|  |  | Ⅵ-2-2 Occupational stress and self-regulation for thoracic surgery specialist nurses | ① | □ | □ | □ | □ | □ |  |
|  |  | Ⅵ-2-3 Professional spirit and social responsibility for thoracic surgery specialist nurses | ① | □ | □ | □ | □ | □ |  |
|  |  | Ⅵ-2-4 Professional development and career planning for thoracic surgery specialist nurses | ① | □ | □ | □ | □ | □ |  |
|  |  | **Third-level indicators to be added:** | | | | | | | |

If you have any other suggestions, please specify: ____________________________________________________________________________

**Table 4 Organizational management for thoracic surgery specialist nurses**

| **First-level indicators** | **Second-level indicators** | **Importance rating** | | | | | **Modification suggestions** |
| --- | --- | --- | --- | --- | --- | --- | --- |
|  |  | **5** | **4** | **3** | **2** | **1** |  |
| Ⅰ Training paradigm | Ⅰ-1 Total course hours: 480 hours (45 minutes per hour) | □ | □ | □ | □ | □ |  |
|  | Ⅰ-2 Integrating theoretical knowledge and practical skills | □ | □ | □ | □ | □ |  |
|  | Ⅰ-3 Duration: 3 months, comprising 1 month of theoretical learning and 2 months of practical training | □ | □ | □ | □ | □ |  |
|  | Ⅰ-4 Full-time commitment without work responsibilities | □ | □ | □ | □ | □ |  |
|  | **Indicators to be added:** | | | | | | |
| Ⅱ Admission criteria for trainees | Ⅱ-1 Holding a valid nursing practice qualification certificate | □ | □ | □ | □ | □ |  |
|  | Ⅱ-2 Bachelor’s degree or higher education level | □ | □ | □ | □ | □ |  |
|  | Ⅱ-3 Professional title of Nurse Practitioner or higher | □ | □ | □ | □ | □ |  |
|  | Ⅱ-4 At least 6 years of clinical nursing experience | □ | □ | □ | □ | □ |  |
|  | Ⅱ-5 At least **3 years** of nursing experience in thoracic surgery | □ | □ | □ | □ | □ |  |
|  | Ⅱ-6 At least 2 years of clinical nursing teaching experience | □ | □ | □ | □ | □ |  |
|  | Ⅱ-7 Basic English proficiency for academic learning | □ | □ | □ | □ | □ |  |
|  | Ⅱ-8 Demonstrated high ethical standards and professional morality | □ | □ | □ | □ | □ |  |
|  | **Indicators to be added:** | | | | | | |
| Ⅲ Faculty selection criteria | Ⅲ-1 Bachelor’s degree or higher (nurses); master’s degree or higher (physicians) | □ | □ | □ | □ | □ |  |
|  | Ⅲ-2 Intermediate professional title or above (nurses); associate senior or higher professional title (physicians) | □ | □ | □ | □ | □ |  |
|  | Ⅲ-3 At least 10 years of specialized experience in related specialties | □ | □ | □ | □ | □ |  |
|  | Ⅲ-4 At leas 5 years of **teaching experience** in academic institutions or clinical training settings | □ | □ | □ | □ | □ |  |
|  | Ⅲ-5 Proven integrity, professional ethics, and dedication to education | □ | □ | □ | □ | □ |  |
|  | **Indicators to be added:** | | | | | | |

If you have any other suggestions, please specify: ____________________________________________________________________________

**Table 5 Evaluation methods for thoracic surgery specialist nurses**

| **First-level indicators** | **Second-level indicators** | **Importance rating** | | | | | **Modification suggestions** |
| --- | --- | --- | --- | --- | --- | --- | --- |
|  |  | **5** | **4** | **3** | **2** | **1** |  |
| Ⅰ Formative evaluation | Ⅰ-1 Class participation | □ | □ | □ | □ | □ |  |
|  | Ⅰ-2 Case analysis | □ | □ | □ | □ | □ |  |
|  | **Ⅰ-3 Chinese/English professional literature reading presentation** | □ | □ | □ | □ | □ |  |
|  | **Ⅰ-4 Health education popularization video/manual development** | □ | □ | □ | □ | □ |  |
|  | **Indicators to be added:** | | | | | | |
| Ⅱ Summative evaluation | Ⅱ-1 Specialty theoretical assessment | □ | □ | □ | □ | □ |  |
|  | Ⅱ-2 Specialty skills assessment: Objective Structured Clinical Examination (OSCE) | □ | □ | □ | □ | □ |  |
|  | Ⅱ-3 Case nursing report | □ | □ | □ | □ | □ |  |
|  | Ⅱ-4 Research proposal | □ | □ | □ | □ | □ |  |
|  | **Indicators to be added:** | | | | | | |

If you have any other suggestions, please specify: ____________________________________________________________________________

**Section 3: Expert familiarity and judgement with the content of the survey**

| ****Familiarity level with research questions**** | □Very familiar  □Relatively familiar  □Moderately familiar  □Somewhat unfamiliar  □Not familiar | ****Degree of judgment on research indicators**** | **Basis for judgment** | ****The degree of impact on your judgment**** | | |
| --- | --- | --- | --- | --- | --- | --- |
|  |  |  |  | **High impact** | **Moderate impact** | **Low impact** |
|  |  |  | Theoretical analysis | □ | □ | □ |
|  |  |  | Practical experience | □ | □ | □ |
|  |  |  | Related domestic and international literature/data | □ | □ | □ |
|  |  |  | Subjective judgment | □ | □ | □ |

**The present survey has now reached its conclusion. Once again, we extend our heartfelt gratitude for your invaluable support throughout this process. May your professional journey be filled with continued success and fulfillment.**
