# Supplementary material for: Construction of the core competencies training system for thoracic surgery specialist nurses: A mixed-methods study
Source: PLoS One. 2026 Feb 2;21(2):e0339777. doi: 10.1371/journal.pone.0339777 (PMC12863540; doi:10.1371/journal.pone.0339777)
Supplement: S1 File — (ZIP) [file pone.0339777.s001.zip › S2 File. Literature quality assessment.zip!/12. 基于成果导向教育理念的军队专科护士培养方案的构建研究(Construction of the cultivation program for specialist nurses in military cardiothoracic surgery department under the concept of outcome-based education).docx]

**JBI critical appraisal tool for quasi-experimental studies**

| **Assessor: Li YJ, Zhu D** | | | | | **Date of Appraisal: 2024.12** | | **Record Number: 12** | | | | | |
| --- | --- | --- | --- | --- | --- | --- | --- | --- | --- | --- | --- | --- |
| **Study Author: Yang J, Duan YZ, Lu XY, Li Y** | | | | | **Study Title: 基于成果导向教育理念的军队专科护士培养方案的构建研究（Construction of the cultivation program for specialist nurses in military cardiothoracic surgery department under the concept of outcome-based education）** | | **Study Year: 2023** | | | | | |
| **Internal Validity** | | | | | | **Choice - Comments/Justification** | | **Yes** | **No** | | **Unclear** | **N/A** |
| **Bias related to temporal precedence** | | | | | | | | | | | | |
| **1** | **It is clear in the study what is the “cause” and what is the “effect” (ie, there is no confusion about which variable comes first)?** | | | | |  | | ☒ | ☐ | | ☐ | ☐ |
| **Bias related to selection and allocation** | | | | | | | | | | | | |
| **2** | **Was there a control group?** | | | | |  | | ☐ | ☒ | | ☐ | ☐ |
| **Bias related to confounding factors** | | | | | | | | | | | | |
| **3** | **Were participants included in any comparisons similar?** | | | | |  | | ☒ | ☐ | | ☐ | ☐ |
| **Bias related to administration of intervention/exposure** | | | | | | | | | | | | |
| **4** | **Were the participants included in any comparisons receiving similar treatment/care, other than the exposure or intervention of interest?** | | | | |  | | ☐ | ☐ | | ☐ | ☒ |
| **Bias related to assessment, detection, and measurement of the outcome** | | | | | | | | | | | | |
| **5** | **Were there multiple measurements of the outcome, both pre and post the intervention/exposure?** | | | | |  | | **Yes** | **No** | | **Unclear** | **N/A** |
|  | **Outcome 1** | | | | | **Expert enthusiasm** | | ☐ | ☐ | | ☐ | ☒ |
|  | **Outcome 2** | | | | | **Expert authority coefficient** | | ☐ | ☐ | | ☐ | ☒ |
|  | **Outcome 3** | | | | | **Expert opinion coordination level** | | ☐ | ☐ | | ☐ | ☒ |
| **6** | **Were the outcomes of participants included in any comparisons measured in the same way?** | | | | |  | | **Yes** | **No** | | **Unclear** | **N/A** |
|  | **Outcome 1** | | | | | **Expert enthusiasm** | | ☒ | ☐ | | ☐ | ☐ |
|  | **Outcome 2** | | | | | **Expert authority coefficient** | | ☒ | ☐ | | ☐ | ☐ |
|  | **Outcome 3** | | | | | **Expert opinion coordination level** | | ☒ | ☐ | | ☐ | ☐ |
| **7** | **Were outcomes measured in a reliable way** | | | | |  | | **Yes** | **No** | | **Unclear** | **N/A** |
|  | **Outcome 1** | | | | | **Expert enthusiasm** | | ☒ | ☐ | | ☐ | ☐ |
|  | **Outcome 2** | | | | | **Expert authority coefficient** | | ☒ | ☐ | | ☐ | ☐ |
|  | **Outcome 3** | | | | | **Expert opinion coordination level** | | ☒ | ☐ | | ☐ | ☐ |
| **Bias related to participant retention** | | | | | | | | | | | | |
| **8** | **Was follow-up complete and if not, were differences between groups in terms of their follow-up adequately described and analyzed?** | | | | |  | |  | | | | |
|  | **Outcome 1** | | | | | **Expert enthusiasm** | | **Yes** | **No** | **Unclear** | | **N/A** |
|  |  | Result 1 | | | | Expert enthusiasm | | ☒ | ☐ | | ☐ | ☐ |
|  | **Outcome 2** | | | | | **Expert authority coefficient** | | **Yes** | **No** | | **Unclear** | **N/A** |
|  |  | Result 1 | | | | Expert authority coefficient | | ☒ | ☐ | | ☐ | ☐ |
|  | **Outcome 3** | | | | | **Expert opinion coordination level** | | **Yes** | **No** | | **Unclear** | **N/A** |
|  |  | Result 1 | | | | Expert opinion coordination level | | ☒ | ☐ | | ☐ | ☐ |
| **Statistical Conclusion Validity** | | | | | | **Choice - Comments/Justification** | |  |  | |  |  |
| **9** | **Was appropriate statistical analysis used?** | | | | |  | |  | | | | |
|  | **Outcome 1** | | | | | **Expert enthusiasm** | | **Yes** | **No** | | **Unclear** | **N/A** |
|  |  | Result 1 | | | | Expert enthusiasm | | ☒ | ☐ | | ☐ | ☐ |
|  | **Outcome 2** | | | | | **Expert authority coefficient** | | **Yes** | **No** | | **Unclear** | **N/A** |
|  |  | Result 1 | | | | Expert authority coefficient | | ☒ | ☐ | | ☐ | ☐ |
|  | **Outcome 3** | | | | | **Expert opinion coordination level** | | **Yes** | **No** | | **Unclear** | **N/A** |
|  |  | Result 1 | | | | Expert opinion coordination level | | ☒ | ☐ | | ☐ | ☐ |
| **Overall appraisal:** | | | **Include: ☒** | **Exclude: ☐** | | **Seek Further Info: ☐** | | | | | | |
| **Comments: This study is a moderate-quality literature, and after group discussion, we have unanimously decided to include it in our research.** | | | | | | | | | | | | |
