# Supplementary material for: Construction of the core competencies training system for thoracic surgery specialist nurses: A mixed-methods study
Source: PLoS One. 2026 Feb 2;21(2):e0339777. doi: 10.1371/journal.pone.0339777 (PMC12863540; doi:10.1371/journal.pone.0339777)
Supplement: S1 File — (ZIP) [file pone.0339777.s001.zip › S2 File. Literature quality assessment.zip!/8. 胸心外科专科护士培养模式初步分析(Preliminary analysis of nurse training mode in thoracic and cardiac Surgery).docx]

JBI Critical Appraisal Checklist for
Qualitative Research

Reviewer	_Li YJ, Zhu D___________________________ Date__2024.12________________________

Author_Ren J_	__ Year__2018______ Record Number___8___

|  | Yes | No | Unclear | Not applicable |
| --- | --- | --- | --- | --- |
| 1. Is there congruity between the stated philosophical perspective and the research methodology? | ☑ | □ | □ | □ |
| 2. Is there congruity between the research methodology and the research question or objectives? | ☑ | □ | □ | □ |
| 3. Is there congruity between the research methodology and the methods used to collect data? | ☑ | □ | □ | □ |
| 4. Is there congruity between the research methodology and the representation and analysis of data? | ☑ | □ | □ | □ |
| 5. Is there congruity between the research methodology and the interpretation of results? | ☑ | □ | □ | □ |
| 6. Is there a statement locating the researcher culturally or theoretically? | □ | ☑ | □ | □ |
| 7. Is the influence of the researcher on the research, and vice- versa, addressed? | ☑ | □ | □ | □ |
| 8. Are participants, and their voices, adequately represented? | ☑ | □ | □ | □ |
| 9. Is the research ethical according to current criteria or, for recent studies, and is there evidence of ethical approval by an appropriate body? | □ | □ | ☑ | □ |
| 10. Do the conclusions drawn in the research report flow from the analysis, or interpretation, of the data? | ☑ | □ | □ | □ |

Overall appraisal:	Include	☑	Exclude	□	Seek further info □

Comments (Including reason for exclusion)：This study is a moderate-quality literature, and after group discussion, we have unanimously decided to include it in our research.
